# Cross-continental comparison of plant reproductive phenology shows high intraspecific variation in temperature sensitivity

**DOI:** 10.1093/aobpla/plae058

**Published:** 2024-10-23

**Authors:** Rachel A Reeb, J Mason Heberling, Sara E Kuebbing

**Affiliations:** Department of Biological Sciences, University of Pittsburgh, 4249 Fifth Ave, Pittsburgh, PA 15260, USA; Section of Botany, Carnegie Museum of Natural History, 4400 Forbes Ave, Pittsburgh, PA 15213, USA; Section of Botany, Carnegie Museum of Natural History, 4400 Forbes Ave, Pittsburgh, PA 15213, USA; Department of Biological Sciences, University of Pittsburgh, 4249 Fifth Ave, Pittsburgh, PA 15260, USA; The Forest School at the Yale School of the Environment, 360 Prospect St, Yale University, New Haven, CT 06511, USA

**Keywords:** climate change, community science, intraspecific, phenological sensitivity, phenology, plants, temperature, warming

## Abstract

The success of plant species under climate change will be determined, in part, by their phenological responses to temperature. Despite the growing need to forecast such outcomes across entire species ranges, it remains unclear how phenological sensitivity to temperature might vary across individuals of the same species. In this study, we harnessed community science data to document intraspecific patterns in phenological temperature sensitivity across the multicontinental range of six herbaceous plant species. Using linear models, we correlated georeferenced temperature data with 23 220 plant phenological records from *iNaturalist* to generate spatially explicit estimates of phenological temperature sensitivity across the shared range of species. We additionally evaluated the geographic association between local historic climate conditions (i.e. mean annual temperature [MAT] and interannual variability in temperature) and the temperature sensitivity of plants. We found that plant temperature sensitivity varied substantially at both the interspecific and intraspecific levels, demonstrating that phenological responses to climate change have the potential to vary both within and among species. Additionally, we provide evidence for a strong geographic association between plant temperature sensitivity and local historic climate conditions. Plants were more sensitive to temperature in hotter climates (i.e. regions with high MAT), but only in regions with high interannual temperature variability. In regions with low interannual temperature variability, plants displayed universally weak sensitivity to temperature, regardless of baseline annual temperature. This evidence suggests that pheno-climatic forecasts may be improved by accounting for intraspecific variation in phenological temperature sensitivity. Broad climatic factors such as MAT and interannual temperature variability likely serve as useful predictors for estimating temperature sensitivity across species’ ranges.

## Teaser

Plants adjust their phenology, or timing of life cycle events, every year in response to annual changes in temperature. As temperatures increase with climate change, the success of plants will be determined, in part, by their innate phenological ‘responsiveness’ to temperature. Despite the growing need to forecast this climate change outcome in plant species, it remains unclear how ‘temperature responsiveness’ might differ across individuals of the same species. Harnessing an exceptional dataset of citizen science observations from multiple continents, we demonstrate that there is significant variation in individual ‘temperature responsiveness’ across species ranges. Furthermore, we provide evidence that plant ‘temperature responsiveness’ can be explained by local historic climate conditions. These findings will contribute to the development of predictive tools which estimate the long-term survival and success of plant species, based on their phenological responsiveness to climate change.

## Introduction

Global climate change poses a significant threat to biodiversity and can catalyse mass species extinctions. To predict and mitigate this threat, scientists and conservationists need to understand how phenotypic traits enable certain species to endure rapid climatic shifts (Román-Palacios and Wiens 2020). The impact of climate change on plant reproduction and growth is greatly determined by phenological traits (Inouye 2022). Phenology, or the timing of life cycle events, delineates the time during which plants germinate or leaf out, reproduce and senesce. Plants time their life cycle according to environmental fluctuations to maximize survival and reproduction by developing for the greatest part under favourable environmental conditions, within a time window also known as the growing season (Rathcke and Lacey 1985; Fenner 1998; Varpe 2017). The temporal boundaries of the growing season are defined by the climatic stressors unique to a given ecosystem. For temperate environments, the growing season is strongly bounded by freezing temperatures in the spring and fall (Rathcke and Lacey 1985; Zohner *et al.* 2017).

Mismatches between a plant’s phenology and the growing season can result in reduced plant fitness. Outside the growing season, plants have limited access to fluctuating seasonal resources, like water or soil nutrients, but also face increased exposure to extreme climatic stressors, like frost and drought (Fenner 1998; Inouye 2000, 2008; Augspurger 2009). The risk derived from phenological mismatches is particularly high in temperate environments where the growing season is limited in length and can vary between years (Mccabe *et al.* 2015). Here, plants rely on a combination of climate and photoperiod cues to track the beginning and end of the growing season (Wolkovich *et al.* 2014; Flynn and Wolkovich 2018).

The ability of an organism to track environmental change by shifting components of its life cycle is measured as ‘phenological sensitivity’, a change in phenological timing that occurs in response to a change in an environmental cue (Thackeray *et al.* 2016). Plants with low sensitivity respond conservatively to changing environmental conditions within their life cycle by exhibiting minimal variation in phenology. Highly sensitive plants respond opportunistically to changing environmental conditions by exhibiting large shifts in phenology. There is widespread variation in sensitivity across species and habitats, reflecting the diverse phenological strategies adapted by plants to adjust to a variety of conditions (Flynn and Wolkovich 2018; Park *et al.* 2018; Kopp *et al.* 2020). A ‘Phenological strategy’ encompasses the suite of traits that dictate the phenological timing of an organism (Pau *et al.* 2011; Tang *et al.* 2016). This includes the baseline (mean) timing of phenology, coupled with its sensitivity to various environmental cues. In temperate climates, seasonal temperature cues tend to be the primary driver of plant phenological change, relative to the effects of precipitation and photoperiod (Wolkovich *et al.* 2013; Wang *et al.* 2020).

Temperature sensitivity is thus an important indicator of temperate plant species’ phenological responses to climate change (Calinger *et al.* 2013; Wang *et al.* 2020). As such, scientists have made extensive efforts to document the standing variation in temperature sensitivity across species and forecast the fitness outcomes of different phenological strategies under future climate conditions (Morin *et al.* 2009; Piao *et al.* 2019; Iler *et al.* 2021). However, such estimates of temperature sensitivity have historically been generalized to the species level, with studies relying on measurements from a sample study region as a proxy for temperature sensitivity across the entire species range (Chuine and Beaubien 2001; Morin *et al.* 2009; Thackeray *et al.* 2016). This assumption is likely over-simplified, as it ignores the probability that phenological strategies vary among individuals of the same species (Diez *et al.* 2012; Xie *et al.* 2022; Zettlemoyer and Peterson 2021). The extent to which temperature sensitivity in phenology varies intraspecifically remains understudied and limits our understanding of climate change outcomes at larger scales (Gutiérrez and Wilson 2021; Love and Mazer 2021; Pearson *et al.* 2021). Variations in the magnitude of temperature sensitivity could generate uneven phenological responses to climate change across the range of species (Des Roches *et al.* 2018; Gutiérrez and Wilson 2021).

There is a need to identify intraspecific variation in temperature sensitivity across species ranges, as well as identify metrics that are useful for predicting such variation. Intraspecific variation in phenotypic traits can arise via neutral evolutionary processes, such as genetic drift, but also in response to the local environment, via plasticity and adaptation (Albert *et al.* 2011). Because of the tight link between phenological traits and fitness, phenological strategies are likely to be shaped by local adaptation and plastic responses to local growing season conditions (Park and Post 2022). Unfortunately, direct tests of local adaptation are complex to execute over large spatiotemporal scales and across multiple species. However, establishing the presence of correlations between phenotypic and environmental variation is a powerful tool to identify possible environmental drivers of variation in phenological strategies across wide spatial and temporal scales (Wolkovich *et al.* 2014). A growing number of studies suggest that intraspecific temperature sensitivity may be predicted by two climatic measurements of the growing season: first, mean annual temperature (MAT), which is the long-term average of annual temperature; second, interannual variability in temperature (IVT), which is the standard deviation of long-term annual temperature (Zohner *et al.* 2017; Hitsman and Simons 2020).

Plant phenological strategies are often correlated with MAT. In the USA, for example, various regional studies of reproductive phenology in herbaceous, woody and aquatic growth forms have found that plants exhibit weakened sensitivity to MATs in colder climates at higher latitudes and elevations (Park *et al.* 2018; Hitsman and Simons 2020; Love and Mazer 2021; Xie *et al.* 2022). Weakened temperature sensitivity may act as a conservative bet-hedging strategy for temperate plants living in cold climates (Park *et al.* 2018; Hitsman and Simons 2020). Under this scenario, for example, a delay in phenology due to a cold spring could reduce the likelihood that a plant completes its life cycle within an already short growing season (Zohner *et al.* 2020). In warmer climates, plants may instead optimize their phenological timing through heightened temperature sensitivity (Pau *et al.* 2011). Under this scenario, the growing season is extended relative to cool climates, so that plants can afford more flexibility in the potential timing of their life cycle (Park *et al.* 2018; Hitsman and Simons 2020). Opportunistic phenological tracking, such as earlier leaf-out and reproduction in warmer years, can provide various fitness advantages to plants. In multispecies communities, for example, advanced phenological timing often allows plants to optimize their access to seasonal resources and minimize competitive exclusion by surrounding community members (Grainger *et al.* 2018; Blackford *et al.* 2020; Wolkovich and Donahue 2021).

Phenological strategies can also be correlated with IVT. High IVT causes the window of the growing season to vary unpredictably across years and increases plants’ exposure to extreme climate events such as late-season frost (Giesecke *et al.* 2010). Plants may adopt unique phenological strategies to tolerate such unpredictability in the climate (Zohner *et al.* 2017; Bauer *et al.* 2020). For example, Zohner *et al.* (2017) found that tree species in Eurasia exhibit longer leaf-out periods than in North America, also characterized by higher IVT. In accordance with these patterns, strong temperature sensitivity may also be retained by plants in high-IVT climates, as it allows plants to sense and track stochastic temporal variation in the growing season. In regions with low IVT, temperature sensitivity may have lower benefits and plants in these climates may have weak temperature sensitivity (Pau *et al.* 2011).

In this study, we harnessed a massive repository of community science plant observations to achieve the following: (i) describe spatial (i.e. latitudinal and continental) variation in historical temperature metrics (MAT and IVT) and the phenological records of six plant species in Eurasia and North America; (ii) measure the geographic association between historic climate factors (i.e. MAT and IVT) and intraspecific levels of temperature sensitivity; and (iii) compare interspecific and intraspecific variation in temperature sensitivity. We measured ‘temperature sensitivity’ as the effect of recent temperature on the Julian date of phenological observations. To account for the potential effect of other environmental factors on phenology, we also measured plants’ phenological sensitivity to precipitation and evaluated potential intraspecific variation in this trait.

In the present study, six herbaceous species were used: *Tussilago farfara* L. (Asteraceae)*, Ficaria verna* L. (Ranunculaceae)*, Alliaria petiolata* (Bieb.) Cavara & Grande (Brassicaeae)*, Cirsium arvense* L. (Asteraceae)*, Daucus carota* L. (Apiaceae), and *Lythrum salicaria* L. (Lythraceae). They are all present across both Eurasia (as native) and North America (as non-native) and occupy similar latitudinal ranges. However, they are diverse in taxonomy, habitat type, and life history [**see Supporting Information—****Table S1****]**. Common intraspecific trends in temperature sensitivity are thus more likely to reflect a phenotypic response to species’ shared climate conditions, rather than a response to unshared environmental conditions (such as soil composition or biotic interactions) or random genetic drift. Additionally, wide-ranging species are often characterized as having above-average intraspecific trait variation, as a product of their expanded ecological breadth (Sides *et al.* 2014). The species chosen for this study thus provide a useful system to capture what is likely to be the upper boundary of potential intraspecific variation in the temperature sensitivity of temperate species.

## Materials and Methods

### Data retrieval: phenology

We leveraged the expansive growth of public, digital and community science records that track plant observations across broad spatial areas (Taylor *et al.* 2019). Specifically, we curated 23 220 plant images from *iNaturalist* that had geolocated records of reproductive phenology for all six species (*T. farfara, F. verna, A. petiolata, C. arvense, D. carota* and *L. salicaria*; iNaturalist 2021). Species were selected using the following criteria: (i) occurring in Eurasia and North America; (ii) containing at least 6000 research-grade images on *iNaturalist* between the years 2017 and 2019 (observations in *iNaturalist* are considered ‘research-grade’ when at least 2/3 of community users agree on the species identification); (iii) possessing buds, flowers, and fruits that are easily identifiable in community science images; and (iv) possessing a constrained annual flowering period. After this, the final study species were selected to represent a diversity of characteristics including family, habitat, life history, seasonal timing of reproduction and time and reason of introduction in North America [**see Supporting Information—****Table S1**].

For each species, we downloaded all research-grade images available between 2017 and 2019. From this, we selected a random subset of 5000 images per species (2500 images from each continent) for phenological annotation (except for *A. petiolata,* for which we annotated 3240 images in Eurasia and 3703 images in North America), totalling to 31 943 annotated images. Most of the unannotated records (those that hold a CC-BY-NC license) are also available on GBIF.org (GBIF.orgA 2021; GBIF.orgB 2021; GBIF.orgC 2021; GBIF.orgD 2021; GBIF.orgE 2021; GBIF.orgF 2021).

One of us (RR) annotated the phenophase of images using a discrete four-stage classification scheme that included vegetative, budding, flowering and fruiting stages. We classified images as ‘vegetative’ if no reproductive parts were present, ‘budding’ if one or more unopened flower buds were present, ‘flowering’ if at least one opened flower was present, and ‘fruiting’ if at least one fully formed fruit was present. If there was more than one type of reproductive organ on the plant, the image was labelled based on the later phenophase (e.g. if both flowers and fruits were present, the image was classified as fruiting; Reeb *et al.* 2022). Observations were systematically excluded from the final analysis based on the following conditions: images missing geographic coordinates or observation date (*n* = 282); images that could not be annotated (primarily, these were missing visible reproductive structures and we could not determine the phenophase with certainty; *n* = 2492); images of vegetative phenology (multiple species in this study do not reproduce in the first year and/or have overwintering rosette leaves, causing this unconstrained phase to be an inaccurate data source for measuring temperature sensitivity; *n* = 5421); observations from Iceland, Azores, eastern Asia and Australia (these fell outside of the primary range of species and georeferenced images were too sparse to assess sensitivity at the island or continental level; *n* = 528). The final dataset for analysis contained 23 220 observations of reproductive phenology [**see Supporting Information—****Table S2**].

### Data retrieval: climate

We obtained gridded time-series climate data at 0.5° resolution from the Climatic Research Unit (CRU; Harris *et al.* 2020). Based on the geographic coordinates and observation year of *iNaturalist* observations, we extracted monthly mean temperature and precipitation values from January to May of the observation year. These data were used to calculate standardized temperature and standardized precipitation at each observation site (see ‘Phenological and climatic variables’). We next extracted the monthly minimum temperature for all available months across the 120-year time period (1901–2021) in the CRU dataset (Harris *et al.* 2020). These data were used to calculate long-term values of MAT and IVT at each observation site (see section below). Mean, minimum and maximum monthly temperature values are extremely correlated at this spatial scale and our choice to use one over the other was arbitrary.

### Phenological and climatic variables

The day of year (DOY) of phenology was calculated based on the Julian date (1–365) of the iNaturalist record. We followed standard practice by assuming that the observation date of an opportunistic phenological record reflects the peak date of that event (Primack *et al.* 2004; Ramirez-Parada *et al.* 2022). While there is some inherent measurement uncertainty in the peak date of these events, a large body of work (including Xie *et al.* 2022; Pearson *et al.* 2021; Primack *et al.* 2004, and more) demonstrates that raw phenological observations can accurately be employed to predict temperature sensitivity in phenology. Furthermore, opportunistic data sources are demonstrated to provide comparable estimates of phenological temperature sensitivity to direct monitoring in the field (Davis *et al.* 2015; Ramirez-Parada *et al.* 2022; Zettlemoyer *et al.* 2022).

Temperature and precipitation sensitivity (i.e. the slope of the effect of a given climate cue on the DOY of a phenological event) were measured based on standardized metrics of temperature and precipitation, respectively. Our study includes a combination of species that reproduce in the spring (*T. farfara, F. verna* and *A. petiolata*) and the summer (*C. arvense, D. carota and L. salicaria*). Previous studies have shown that plant reproductive phenology tends to be most sensitive to climate conditions directly preceding the phenological event (Lu *et al.* 2006; Kopp *et al.* 2020). Thus, to calculate climate sensitivity, temperature and precipitation were standardized: (i) based on mean-centred winter climate values (January–March) for the spring-blooming species and (ii) based on mean-centred spring climate values (March–May) for the summer-blooming species [**see Supporting Information—****Fig. S1**]. We calculated MAT as the mean annual minimum temperature (°C) over the 120-year time period. We calculated IVT as the standard deviation (s.d.) of mean annual minimum temperature across the 120-year time period (Zohner *et al.* 2017).

### Statistical analysis

#### Overview

Our analysis relied on a total of four linear models to meet our three study aims. Our first study aim was to describe geographic variation across the study range in three distinct response variables (Aim 1): MAT, IVT and phenology. To do so, we implemented separate models for each of the three response variables. We then used a single, global model to test the remaining two study aims that focussed on understanding the climatic drivers of temperature sensitivity (Aim 2) and comparing interspecific and intraspecific variation in temperature sensitivity (Aim 3).

We interpret models with higher-order interactions by employing the ‘emmeans’ package in R (Lenth 2021; Battlay 2023; Li *et al.* 2023; Mooney *et al.* 2023; Sinclair *et al.* 2022). We do not discount the challenges in interpreting models with multiple interaction terms; in many instances, testing higher-order interactions is not possible because of limited statistical power to conduct multiple comparisons. However, due to the size of our community science dataset (*n* = 23 220), we are uniquely afforded the opportunity to do so. As we explain in more detail below for each Aim, the ‘emmeans’ package can be used to extract two types of marginal estimates from the global model: (i) the mean value of the response variable at fixed levels of the independent variables and (ii) the simple slope (i.e. effect size) of a numeric independent variable over different levels of the moderating (interacting) variables. *Post hoc* pairwise comparisons, using Tukey’s HSD *P*-value adjustment to correct for multiple comparisons, can then be employed to statistically compare marginal estimates and measure the effect of higher-order interactions. Any independent variable from the full model that is not specified in a *post hoc* analysis is statistically controlled for the purpose of that analysis. In other words, the *post hoc* results tell us the average effect of specified independent variables and modifier (interacting) variables, while accounting for variation caused by other unspecified variables in the model.

#### Describing spatial variation in historical climate metrics (MAT and IVT) and phenology records across continents and latitude (Aim 1)

To assess spatial variation (i.e. continental and latitudinal differences) in the 120-year average climate metrics within the study range, we ran two linear additive models (MAT or IVT ~ continent + latitude). We then conducted *post hoc* pairwise comparisons to estimate differences in MAT and IVT between continents (North America or Eurasia) and latitude.

To assess spatial variation (i.e. continental and latitudinal differences) in reproductive phenology (expressed as DOY) within the study range, we next used a linear model with the following predictors: phenophase (budding, fruiting or flowering), continent (Eurasia or North America), latitude and species (*T. farfara, F. verna, A. petiolata, C. arvense, D. carota* and *L. salicaria*). To assess spatial variation in the three reproductive phenophases, we included phenophase as a two-way interaction term with continent and latitude, respectively. We included an additional a two-way interaction term between phenophase and species to account for species-level differences in phenology. Our model structure was DOY ~ (phenophase × continent) + (phenophase × latitude) + (phenophase × species). We then conducted *post hoc* pairwise comparisons to assess differences in phenological timing across phenophases, continents and latitude. Results from this *post hoc* analysis were averaged across species.

#### Global model selection (Aims 2 and 3)

We evaluated several potential model structures that would allow us to test Aims 2 and 3 as well as maximize model fit based on AIC selection criterion [**see Supporting Information—****Table S3**]. To measure temperature sensitivity (i.e. the slope of the effect of standardized temperature on observed phenology), we included DOY as the response variable and standardized temperature as an independent variable in the model (; Park *et al.* 2018; Love and Mazer 2021). To evaluate how MAT and IVT modify intraspecific temperature sensitivity in plants (Aim 2), we included a three-way interaction term between standardized temperature, MAT, and IVT in the model. To compare intra- and interspecific variation in temperature sensitivity (Aim 3), we also included a two-way interaction term between ‘species’ and ‘standardized temperature’ in the model.

We included additional independent variables that maximized model fit based on AIC selection criterion [**see Supporting Information—****Table S3**]. We found that model fit was significantly improved by including phenophase (budding, flowering, and fruiting), standardized precipitation and their interactions with MAT and IVT. We also found that model fit was improved by including interaction terms between species and all existing model terms. The best fit model was as follows:


$$\begin{array}{l} \begin{array}{llll} & DOY(standardizedtemperature\times MAT\;\times \;IVT\times species)\; & +(standardizedprecipitation\times MAT\times IVT\times species)\; & +(phenophase\times MAT\times IVT\times species).\; \end{array} \end{array}$$

The presence of significant three and four-way interactions in the best fitting model indicates that plant phenological timing is co-dependent on species’ identity, phenophase, contemporary environmental cues (i.e. standardized temperature and precipitation), and historic environmental variables (i.e. MAT and IVT), which is well-established in the phenological literature (Flynn and Wolkovich 2018; Park *et al.* 2018). As described in detail below, specific results for Aims 2 and 3 were produced by performing *post hoc* analyses on the global model.

#### Measuring the geographic association between MAT, IVT and intraspecific temperature sensitivity (Aim 2)

To test our second study aim, we performed a *post hoc* analysis on the global model that estimated intraspecific trends in temperature sensitivity across climatic gradients of MAT and IVT. We conducted a *post hoc* analysis on the following subset of terms: DOY ~ (standardized temperature × MAT × IVT). Temperature sensitivity was measured as the marginal estimated slope (i.e. effect size) of standardized temperature. We evaluated how MAT and IVT modify intraspecific temperature sensitivity by extracting the marginal slope of standardized temperature for each unique combination of MAT and IVT in the study area (iterated at 0.2 °C intervals for MAT, ranging from −5 to + 15 °C, and iterated at 0.05 s.d. intervals for IVT, ranging from 0.4 to 1.2 s.d.). Marginal estimates were averaged across species and phenophases and controlled for the effect of standardized precipitation. We then conducted pairwise comparisons of the marginal estimated slopes to statistically evaluate how temperature sensitivity changes across climatic gradients in MAT and IVT. Using the results of this *post hoc* analysis, we created a range map of estimated temperature sensitivity by assigning the marginal estimated slope of standardized temperature to each original iNaturalist observation, based on their associated MAT and IVT. Observations were then plotted onto a map based on their original geographic coordinates and assigned a colour scale value to reflect temperature sensitivity.

#### Comparing interspecific and intraspecific variation in phenological temperature sensitivity across the study range (Aim 3)

To test our third study aim, we conducted two *post hoc* analyses that estimated the magnitude of intraspecific and interspecific variation in temperature sensitivity. First, to quantify the magnitude of intraspecific variation, we conducted a *post hoc* analysis on the following subset of terms from the global model: DOY ~ (standardized temperature × MAT × IVT). We iteratively extracted temperature sensitivity (i.e. the marginal estimated slope of standardized temperature) for each unique combination of MAT and IVT in the study area (iterated at 1 °C intervals for MAT ranging from −5 to + 15 °C, and iterated at 0.1 s.d. intervals for IVT, ranging from 0.4 to 1.2 s.d.). *Post hoc* results were averaged across species and phenophases and controlled for the effect of standardized precipitation. We then measured the difference between the maximum and minimum temperature sensitivity within the study area. Second, to quantify the magnitude of interspecific variation in temperature sensitivity, we conducted a *post hoc* analysis on the following subset of terms from the global model: DOY ~ (standardized temperature × species). We extracted the mean temperature sensitivity, (i.e. the marginal estimated slope of standardized temperature) for each species in the study. *Post hoc* results were averaged across phenophases and controlled for the effects of standardized precipitation, MAT and IVT. We then measured the difference between the maximum and minimum temperature sensitivity of study species.

Supplementary to our study aims, we also conducted *post hoc* analyses that measured (i) intraspecific patterns in temperature sensitivity for each individual species and (ii) mean intraspecific patterns in precipitation sensitivity. Detailed methods and results for these supplementary analyses are available in the Supplement. [**see Supporting Information—**Methods S1 and S2; Tables S5, S6, S7, S8, and S9; Figs. S2 and S3].

Residual diagnostics revealed some irregularity in the residuals of the linear model. Upon evaluation of the data, this primarily appears to be caused by the leptokurtic distribution of the response variable (DOY), defined as having fewer values located in the tails than expected under a normal distribution. Three species (*A. petiolata, T. farfara* and *F. verna)* had irregular response variable distribution. To our knowledge, there are no common data transformations to correct leptokurtic normality errors. However, gaussian models tend to be robust to non-normality, particularly when sample sizes are large, and bears a relatively lower risk of type I error than nongaussian models (Knief and Forstmeier 2021). Additionally, non-gaussian and non-parametric models are limited in their ability to test for multi-way interactions. Given the limitations of nongaussian models and the extremely large sample size (*n* = 23 220) of this analysis, we concluded that a linear model with the assumption of a gaussian distribution was the most suited for our dataset.

All data extraction, data cleaning, linear analysis, model validation, and data visualization was performed in R version 4.04, using the ‘dplyr’, ‘raster’, ‘ncdf4’, ‘emmeans’, ‘sjPlot’, ‘ggplot2’, ‘lme4’, ‘DHARMa’, ‘ggmap’, ‘RColorBrewer’, and ‘tidyverse’ packages (Kahle and Wickham 2013; Neuwirth 2014; Bates *et al.* 2015; Pierce 2019; Wickham *et al.* 2019, 2022; Hartig 2020; Hijmans 2020; Lenth 2021; Lüdecke 2021; R Core Team 2021).

## Results

All results reported below reflect marginal estimates or pairwise comparisons of marginal estimates, which are derived from *post hoc* analyses of the linear models. Estimates reported here are statistically significant (*P* < 0.05) unless otherwise indicated.

### Measuring geographic variation in historical MAT, IVT and phenology across continents and latitudes (Aim 1)

**Figure 1.**
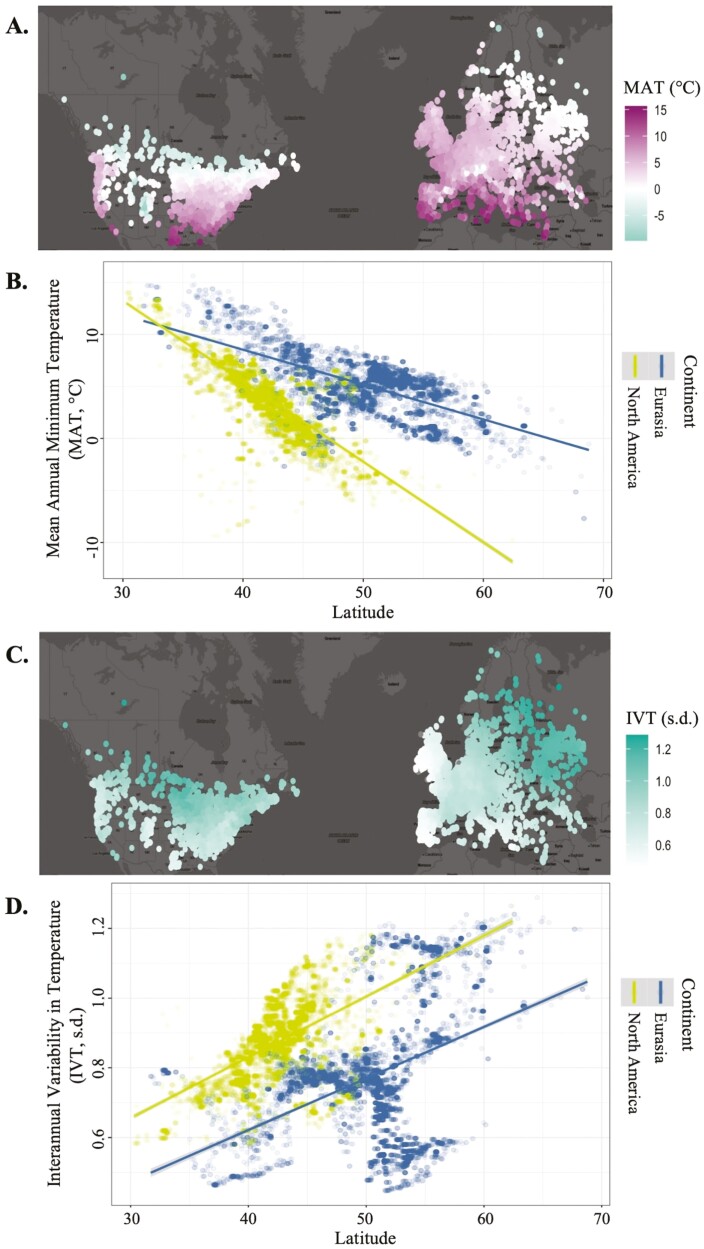
Geographic variation in MAT and IVT, calculated over a 120-year period (1901–2021) for 23 220 iNaturalist observations of six herbaceous plant species. (A) Map of MAT values across the study range. (B) Linear trend in MAT across a latitudinal gradient and continents. (C) Map of IVT values across the study range. (D) Linear trend in IVT across a latitudinal gradient and continents.

On average, Eurasia had a warmer and less variable climate than North America over the 120 year historic temperature period. Mean annual temperatures (measured in °C) in Eurasia were an average of 4.91 °C (± 0.04 s.e.) warmer than in North America. Interannual variability in temperature (measured in s.d.) was 25% lower in Eurasia (-0.22 s.d. ± 0.003 s.e.) than North America. Across both continents, MAT cooled by −0.45°C/°latitude (± 0.003 s.e.) and IVT increased by 0.02 s.d./°latitude (± 0.0002 s.e.; Fig. 1).

Across all species and reproductive phenophases (budding, flowering, fruiting), phenological events occurred later at higher latitudes (+ 0.49 days/°latitude,± 0.07 s.e.). While budding time did not differ between continents (*P* = 0.433) on average, flowering occurred an average of 6.90 days later in North America than Eurasia (± 0.49 s.e.), and fruiting occurred an average of 5.07 days later in North America than Eurasia (± 0.70 s.e.).

### Measuring the geographic association between MAT, IVT and intraspecific temperature sensitivity (Aim 2)

**Figure 2.**
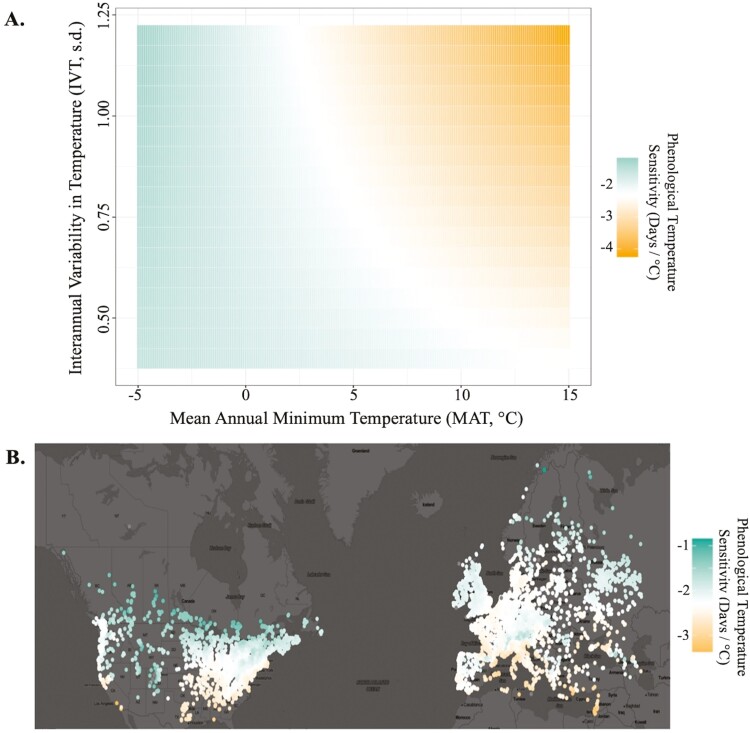
Intraspecific variation in phenological temperature sensitivity across climatic gradients in MAT and IVT. Color scales are centered on the overall average baseline temperature sensitivity (−2.28 days/°C). Temperature sensitivity values are marginal estimated slopes based on the MAT and IVT of a given observation site, extracted from the global model (*n* = 23 220, df = 23 100, adjusted *R*^2^ = 0.817). (A) Heat map of temperature sensitivity across gradients of MAT and IVT. (B) Geographic map of estimated temperature sensitivity across the study range. Points represent geolocated species observations from iNaturalist and color value represents the estimated temperature sensitivity of plants in that location.

In a *post hoc* analysis of the global model, we measured how MAT and IVT modifies intraspecific temperature sensitivity (i.e. the slope of the effect of standardized temperature on phenology DOY) in plants across the study range. Overall, plant phenology was sensitive to local changes in standardized temperature, advancing by a baseline average of −2.28 days/°C (± 0.13 s.e.) (Table 1). We identified substantial intraspecific variation in temperature sensitivity across climatic gradients. Plants exhibited stronger phenological sensitivity to temperature in regions where MAT was historically warmer. Across species, temperature sensitivity strengthened in warmer regions by an average of −0.10/°MAT (± 0.02 s.e.; Table 1). This translates to a 4.4% average baseline deviation in temperature sensitivity per °C change in MAT (Fig. 2). However, the precise geographic association between MAT and temperature sensitivity was modified by local levels of IVT. MAT was a strong predictor of temperature sensitivity in regions with high IVT (−0.16/°MAT ± 0.05 s.e. where IVT = 1.2 s.d.) but its correlation with temperature sensitivity in regions with low IVT was not statistically significant (*P* = 0.690 where IVT = 0.4 s.d.; Table 1, Fig. 2).

**Table 1.** Marginal estimates of temperature sensitivity (measured as the marginal slope of the effect of standardized temperature on phenology DOY) and trends in temperature sensitivity across gradients in MAT and IVT. Marginal estimates of temperature sensitivity are subsampled at MAT levels (−5, 5, and 15°C) and IVT levels (0.4, 0.8, and 1.2 s.d.). The conditional effect of MAT on temperature sensitivity is subsampled at IVT levels (0.4, 0.8, and 1.2 s.d.). Model estimates control for variation in species identity, phenophase and standardized precipitation (*n* = 23 220, df = 23 100 and adjusted *R*^2^ = 0.817). Asterisks indicate a statistically significant *P* value (< 0.05).

MAT (°C)	IVT (s.d.)	Temperature sensitivity (days/°C)	Standard error	*P* value
Overall	Overall	−2.28	0.13	<0.001^**^
−5	0.4	−1.48	0.64	0.019^**^
	0.8	−1.31	0.25	<0.001^**^
	1.2	−1.14	0.48	0.018^**^
5	0.4	−1.92	0.38	<0.001^**^
	0.8	−2.31	0.13	<0.001^**^
	1.2	−2.70	0.34	<0.001^**^
15	0.4	−2.36	0.67	0.001^**^
	0.8	−3.31	0.28	<0.001^**^
	1.2	−4.27	0.64	<0.001^**^
IVT (s.d.)	Effect of MAT on temperature sensitivity (𝚫 temperature sensitivity/°MAT)		Standard error	*P* value
Overall	−0.10		0.02	<0.001^**^
0.4	−0.04		0.05	0.690
0.8	−0.10		0.02	<0.001^**^
1.2	−0.16		0.05	0.002^**^

### Comparing interspecific and intraspecific variation in phenological temperature sensitivity across the study range (Aim 3)

Using *post hoc* analyses of the global model, we compare variation in temperature sensitivity at the intraspecific level (i.e. total variation in temperature sensitivity across the study area) and the interspecific level (i.e. total variation in mean temperature sensitivity across species). We found that temperature sensitivity varied similarly within species as it did among species. Intraspecific estimates of temperature sensitivity ranged between −1.14 days/°C (± 0.48 s.e., where MAT = −5 °C and IVT = 1.2 s.d.) and −4.26 days/°C (± 0.64 s.e., where MAT = 15 °C and IVT = 1.2 s.d.). This translates to 3.12 days/°C of total intraspecific variation in temperature sensitivity across the climatic study area [**see Supporting Information—****Table S4**]. Comparatively, interspecific estimates of temperature sensitivity ranged between −1.07 days/°C (± 0.24 s.e., *A. petiolata)* and −3.37 days/°C (± 0.32 s.e., *T. farfara)*. This translates to 2.30 days/°C of total interspecific variation in temperature sensitivity among the study species [**see Supporting Information—****Table S4**].

### Species-specific differences in temperature sensitivity

While regional MAT and IVT were overarching predictors of temperature sensitivity in every species, we unsurprisingly found that exact climate associations were specific to each species [**see Supporting Information—****Tables S5**  **and**  **S6**, **Figs. S2**  **and**  **S3**]. Full *post hoc* analysis results of species-level temperature sensitivity are available in the supplement [**see Supporting Information—****Tables S5**  **and**  **S6**, **Figs. S2**  **and**  **S3**].

### No intraspecific variation detected in precipitation sensitivity

Plant phenology was slightly sensitive to rainfall, delaying by an average of 0.03 days/mm standardized precipitation (± 0.01 s.e.; []**see Supporting Information—****Table S7]**)]. On average, however, we did not detect intraspecific variation in precipitation sensitivity across the study area [**see Supporting Information—****Tables S7**  **and**  **S8**]. Full *post hoc* results for precipitation sensitivity are available in the supplement [**see Supporting Information—****Table]]]s S7**, **S8**  **and**  **S9**).]]]

## Discussion

### Relationship between the regional climate and plant temperature sensitivity

Harnessing a massive library of community science images, we identified intraspecific patterns in temperature sensitivity throughout the shared multicontinental range of six herbaceous plant species. While plant phenology advanced by an average of −2.28 days/°C, local levels of temperature sensitivity deviated from the baseline by up to ± 1.56 days/°C, or 68%, across the study area. The geographic patterns identified in this study strongly support the hypothesis that a plant’s temperature sensitivity is influenced by local climate conditions. On average, temperature sensitivity strengthened in warmer climates (with high MAT), tracking regional reductions in winter severity and extensions in the length of the growing season (Fig. 2). This finding confirms and extends the patterns found in regional studies of North American flora and demonstrates that clines in temperature sensitivity along climatic gradients are prevalent across a diverse array of temperate habitat types and taxa (Park *et al.* 2018; Hitsman and Simons 2020; Love and Mazer 2021; Xie *et al.* 2022). Uniquely, we found that the localized effect of MAT on plant temperature sensitivity was also contingent upon levels of IVT, suggesting that temperature sensitivity is also influenced by the predictability in the local climate. However, continued study is needed to determine whether the observed patterns in relation to IVT are generalizable to non-herbaceous plant growth forms or life history stages outside of reproduction.

**Figure 3.**
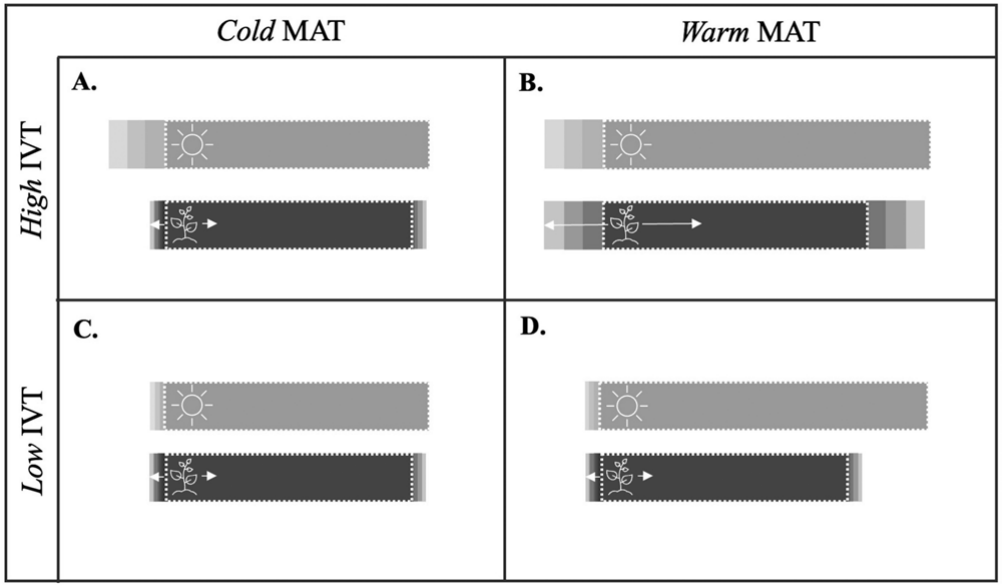
Conceptual diagram of the predicted relationship between regional MAT, IVT and plant temperature sensitivity. Light grey bars depict the hypothetical temporal window of the growing season, a period of time when climate conditions are suitable to support the life cycle of a given species. The average length of the growing season is shortened in cold climates (A and C) and lengthened in warm climates (B and D). In geographic regions with low IVT, the growing season window is consistent from year to year (C and D). In regions with high IVT, the growing season period is unpredictable and varies widely between years (A and B). Dark-grey bars are hypothetical depictions of the minimum temporal window that a species requires to complete its entire life cycle. Possible temporal variation in the phenological window is determined by the temperature sensitivity of plants, with longer arrows depicting plants with stronger temperature sensitivity. Plants with weak temperature sensitivity (i.e. A, C and D) have relatively consistent annual phenological timing regardless of temperature signals, while plants with strong sensitivity (i.e. B) have the potential to exhibit large amounts of variation in phenology in response to temperature signals.

We predict that this pattern reflects a contrast in the phenological strategies adopted by plants in different climatic environments (Fig. 3). In consistent climates (low IVT), plants may exhibit weak temperature sensitivity because they have a reduced need for phenological tracking (Pau *et al.* 2011; Fig. 3C and D). Plants may similarly exhibit weak temperature sensitivity in cold climates (low MAT), to ensure that the life cycle conservatively begins and ends within the narrow frost-free period (Fig. 3A and C). However, strong temperature sensitivity could be uniquely advantageous in climates that are both warm and unpredictable (high MAT and IVT; Pau *et al.* 2011; Fig. 3B). The extended growing season in warm climates allows for flexibility in the potential timing of phenology. In the absence of this constraint, plants may closely track annual changes in temperature in order to optimize their access to seasonal resources and gain competitive advantages over other species (Blackford *et al.* 2020; Wolkovich and Donahue 2021). Our primary finding (that temperature sensitivity is concurrently dependent on regional levels of MAT and IVT) may partially explain the incongruencies among previous studies that estimate temperature sensitivity trends across gradients in MAT alone (Cook *et al.* 2012; Prevéy *et al.* 2017; Park *et al* 2018; Love and Mazer 2021; Xie *et al.* 2022). Continued exploration will be needed to unpack the interactive effects of other temperature cues, such as winter chilling or end-of-season temperature, and additional environmental cues, such as photoperiod, on intraspecific levels of temperature sensitivity.

We found strong overarching patterns of temperature sensitivity among the six species included in this study, which differed in habitat, life history traits and phylogenetic characteristics but shared a common climate across their multicontinental ranges [**see Supporting Information—**Table S1]. Main trends across diverse species are likely driven by a non-random phenotypic response to climatic variation. Whether these phenotypic responses are a function of trait plasticity, evolution or both is unclear. Further experimental research could determine the mechanisms responsible for the observed spatial association between MAT, IVT and plant temperature sensitivity. Common garden and reciprocal transplant experiments can test the relative contributions of plasticity and local adaptation to temperature sensitivity across climatic gradients (Zettlemoyer and Peterson 2021).

### Implications for pheno-climatic forecasts and conservation

Anthropogenic global warming may reduce the effectiveness of some plant phenological strategies over time. In many regions, warming will advance the start date and increase the length of the growing season window (Christiansen *et al.* 2011). Researchers predict that plants with strong temperature sensitivity will have a robust capacity to track such shifts in the growing season and succeed under climate change (Cleland *et al.* 2012). In other situations, however, warming may increase unpredictable weather anomalies like false springs or early fall frosts (Inouye 2008; Augspurger 2013). Increases in interannual temperature variability could degrade the reliability of temperature signals as indicators of the growing season window and disadvantage plants with strong temperature sensitivity (Ma *et al.* 2019).

Pheno-climatic forecasts, which model phenological responses to climate, will be useful tools for predicting the success of species under climate change. Our study findings suggest that the accuracy of existing pheno-climatic forecasts may be improved by accounting for intraspecific differences in temperature sensitivity across the species range (Wolkovich *et al.* 2014; Tang *et al.* 2016; Love and Mazer 2021). For some well-observed species—like those studied here—emerging community science datasets will be useful for capturing temperature sensitivity across the entire species range.

Plants that are unable to migrate or adapt to warming may experience population declines in the absence of human intervention (Visser 2008; Nicotra *et al.* 2010; Anderson *et al.* 2012; Ettinger *et al.* 2022). Conservationists may explore the utility of ‘intraspecific variation in temperature sensitivity’ as a predictive metric to identify and prioritize vulnerable species. In this study, we intentionally focussed our assessment on widespread species that occupied a large climatic niche, in order to capture the ‘upper bounds’ of possible intraspecific variation in plant temperature sensitivity. Regardless of range size, species that maintain a large variety of phenological strategies across their populations may be more resilient to climate change (Oney *et al.* 2013). Comparatively, species that have low intraspecific variation in temperature sensitivity may lack this buffer and thus benefit from targeted conservation interventions.

### Strengths and limitations of community science phenological data

The opportunities, limitations and statistical methods for modelling phenology using ‘opportunistic’ data sources (such as community science records or herbarium specimens) have been reviewed comprehensively in the literature (Davis *et al.* 2015; Barve *et al.* 2020; Gallinat *et al.* 2021; Pearson *et al.* 2021). The strength of opportunistic phenological data, when compared to time-series or repeated sampling data, lies in the extensive quantity and spatial coverage of observations across broad environmental gradients (Barve *et al.* 2020; di Cecco *et al.* 2021; Gallinat *et al.* 2021). However, a potential limitation of opportunistic phenological data is the risked introduction of spatial bias, whereby sampling efforts tend to be more concentrated in areas where the community platform (such as iNaturalist) is popularized (Di Cecco *et al.* 2021). We considered this bias in the study design by selecting species whose range was primarily concentrated in North America and western Eurasia, where iNaturalist usage is prevalent, and by sampling an even number of observations on each continent.

## Conclusion

Successful conservation practices in the era of climate change will require an expanded scientific effort to document intraspecific variation in climate-sensitive traits across a wide range of taxa, habitats and continents. Here, we harnessed community science data to document intraspecific patterns of temperature sensitivity and provide evidence for a strong geographic association between regional climate and temperature sensitivity. The modern boom in digital biodiversity records, made available by community science platforms, museums and other academic institutions, provide scientists with an unprecedented opportunity to document intraspecific variation in climate-sensitive traits at the global scale. Continued exploration in this arena will be critical to the development of predictive tools which estimate the success of plant phenological responses to climate change.

## Supporting Information

The following additional information is available in the online version of this article –

**Methods S1.** Methods for *post hoc* analysis that measured intraspecific patterns in temperature sensitivity for each individual species.

**Methods S2.** Methods for post hoc analysis that measured intraspecific patterns in precipitation sensitivity.

**Figure S1.** Reproductive phenology day of year of spring-blooming and summer-blooming species observations across reproductive phenophases (budding, flowering and fruiting).

**Figure S2.** Heat map of species-specific intraspecific variation in phenological temperature sensitivity across climatic gradients in mean annual temperature and interannual variability in temperature.

**Figure S3.** Geographic map of species-specific intraspecific variation in phenological temperature sensitivity.

**Table S1.** Description of life history species traits for six herbaceous plant species included in this study.

**Table S2.** Sample sizes of final dataset across species and continents.

**Table S3.** Model selection for the ‘global model’ to assess intraspecific variation in ‘temperature sensitivity’ (Aims 2 and 3).

**Table S4.** Species-specific estimates of in temperature sensitivity and intraspecific variation in temperature sensitivity.

**Table S5.** Species-specific independent effects of mean annual minimum temperature and interannual variability in temperature on temperature sensitivity.

**Table S6.** Species-specific marginal estimates and contrasts in temperature sensitivity across interacting levels of mean annual temperature and interannual variability in temperature.

**Table S7.** Estimated marginal trends in precipitation sensitivity across interacting levels of mean annual temperature and interannual variability in temperature.

**Table S8.** Estimated marginal trends in precipitation sensitivity across a gradient of mean annual temperature and at different levels of interannual variability in temperature.

**Table S9.** Species-specific estimated marginal trends in precipitation sensitivity.

plae058_suppl_Supplementary_Material

## Data Availability

The data and R scripts that support the findings of this study are openly available in Zenodo at http://doi.org/10.5281/zenodo.7828740.
