# Supplementary material for: Cross-continental comparison of plant reproductive phenology shows high intraspecific variation in temperature sensitivity
Source: AoB Plants. 2024 Oct 23;16(6):plae058. doi: 10.1093/aobpla/plae058 (PMC11639196; doi:10.1093/aobpla/plae058)
Supplement: plae058_suppl_Supplementary_Material [file plae058_suppl_supplementary_material.pdf]

Supplementary Material

Supplementary material for “Cross-continental comparison of plant reproductive phenology shows high intraspecific variation in temperature sensitivity.”

Supplementary Methods

Supplementary Methods S1:

We conducted a post-hoc analysis (using the 'emtrends' function in the emmeans package) that measured intraspecific patterns in temperature sensitivity for each individual species. Temperature sensitivity was again measured as the mean marginal trend in standardized temperature on phenology DOY. We conducted the following post-hoc test on the global model: (DOY ~ standardized temperature * MAT * ITV * species). The conditional effects of MAT, IVT, and species identity on temperature sensitivity were obtained by iteratively extracting the marginal trend of temperature sensitivity across each unique combination of species identity, MAT, and IVT in the study area (iterated at 0.2° intervals for MAT ranging from -5 to +15 °C, and iterated at 0.05 s.d. intervals for IVT, ranging from 0.4 to 1.2 s.d.). These marginal estimates were then used to statistically evaluate species-specific changes in temperature sensitivity across climatic gradients in MAT and IVT. Results of these post-hoc tests were averaged across phenophases and controlled for the effect of standardized precipitation.

Supplementary Methods S2:

We conducted a post-hoc analysis that measured intraspecific patterns in precipitation sensitivity. Precipitation sensitivity was measured as the mean marginal effect of standardized precipitation on phenology DOY. We conducted the following post-hoc test on the global model: (DOY ~ standardized precipitation * MAT * ITV). The conditional effects of MAT and IVT on precipitation sensitivity were obtained by extracting the marginal trend of precipitation sensitivity for each unique combination of MAT and IVT in the study area (iterated at 0.2° intervals for MAT ranging from -5 to +15 °C, and iterated at 0.05 s.d. intervals for IVT, ranging from 0.4 to 1.2 s.d.). These marginal estimates were then used to statistically evaluate changes in precipitation sensitivity across climatic gradients in MAT and IVT. Results of these post-hoc tests were averaged across species and phenophases and controlled for the effect of standardized temperature.

Supplementary Figures

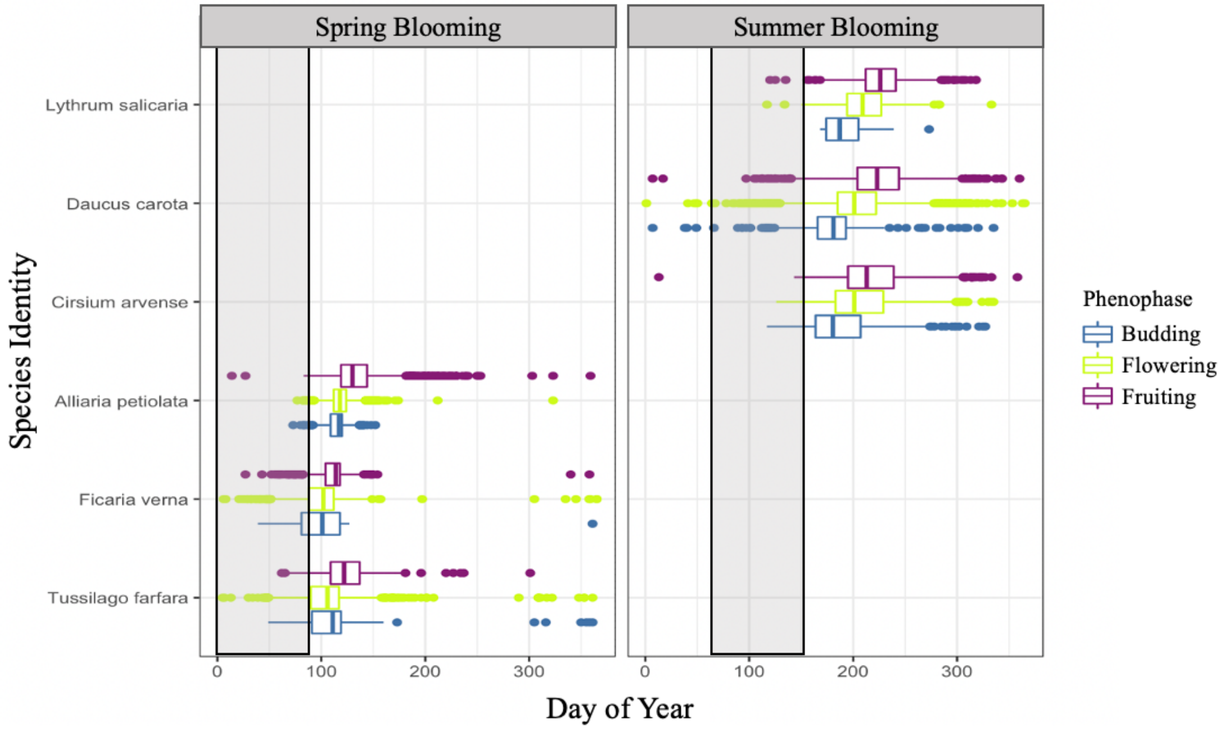

Figure S1 Reproductive phenology day of year (DOY) of spring blooming (left panel) and summer blooming (right panel) species observations across reproductive phenophases (budding, flowering, and fruiting). Boxplot values represents the median and interquartile range of phenology observations. Shaded grey bars represent the 3-month temporal window used to calculate phenological sensitivity to climate for spring and summer blooming species, respectively, using standardized temperature metrices.

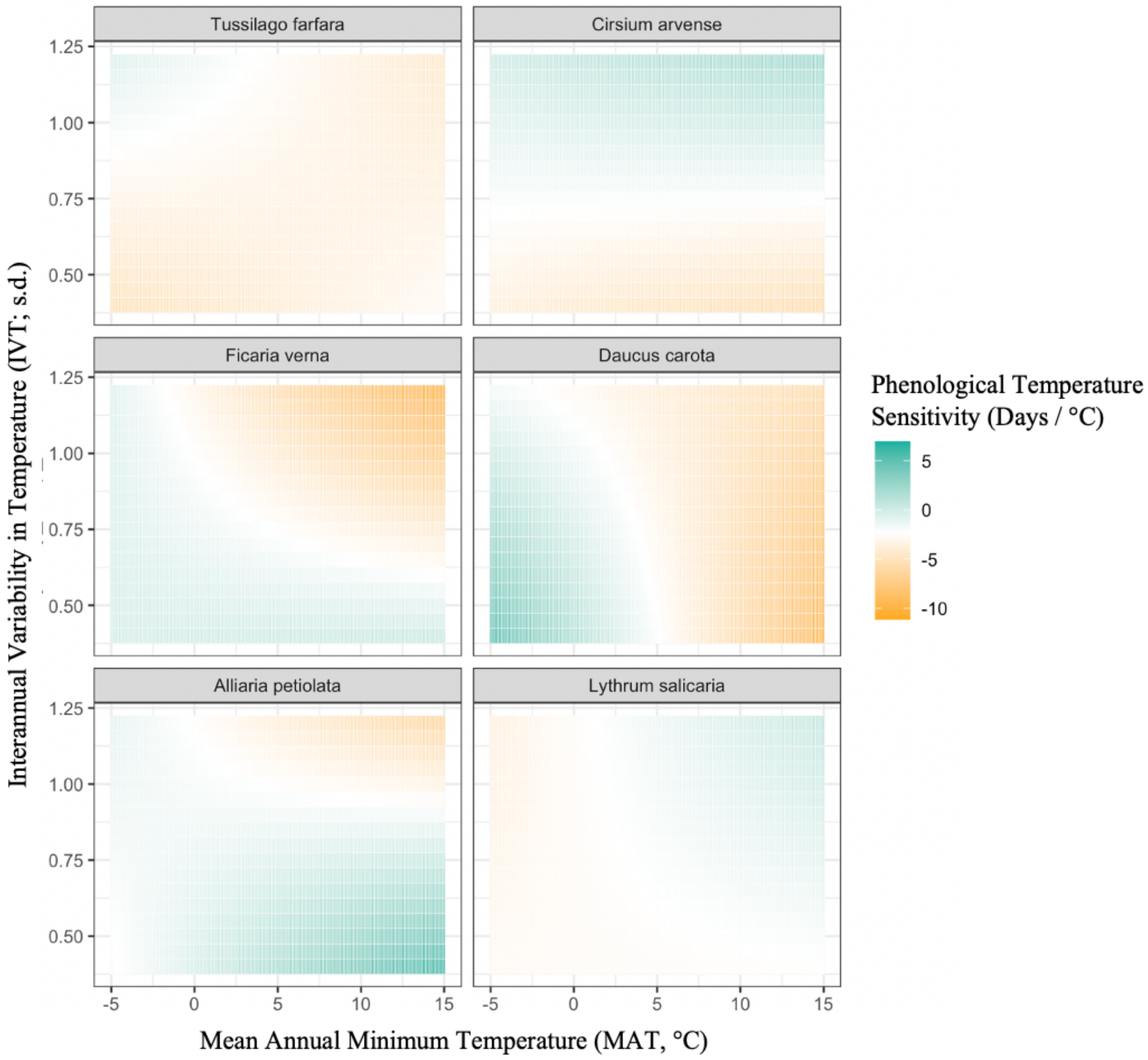

Figure S2. Heat map of species-specific intraspecific variation in phenological temperature sensitivity across climatic gradients in mean annual temperature (MAT) and interannual variability in temperature (IVT). Color scale is centered on the baseline average temperature sensitivity (-2.28 days / °C), with teal indicating a weaker-than-average level of temperature sensitivity and orange indicating a stronger-than-average level of temperature sensitivity. Temperature sensitivity values are estimated marginal trends, extracted across all unique combinations of species identity, MAT, and IVT (n = 23,220, df = 23,100, adjusted $R^2 = 0.817$).

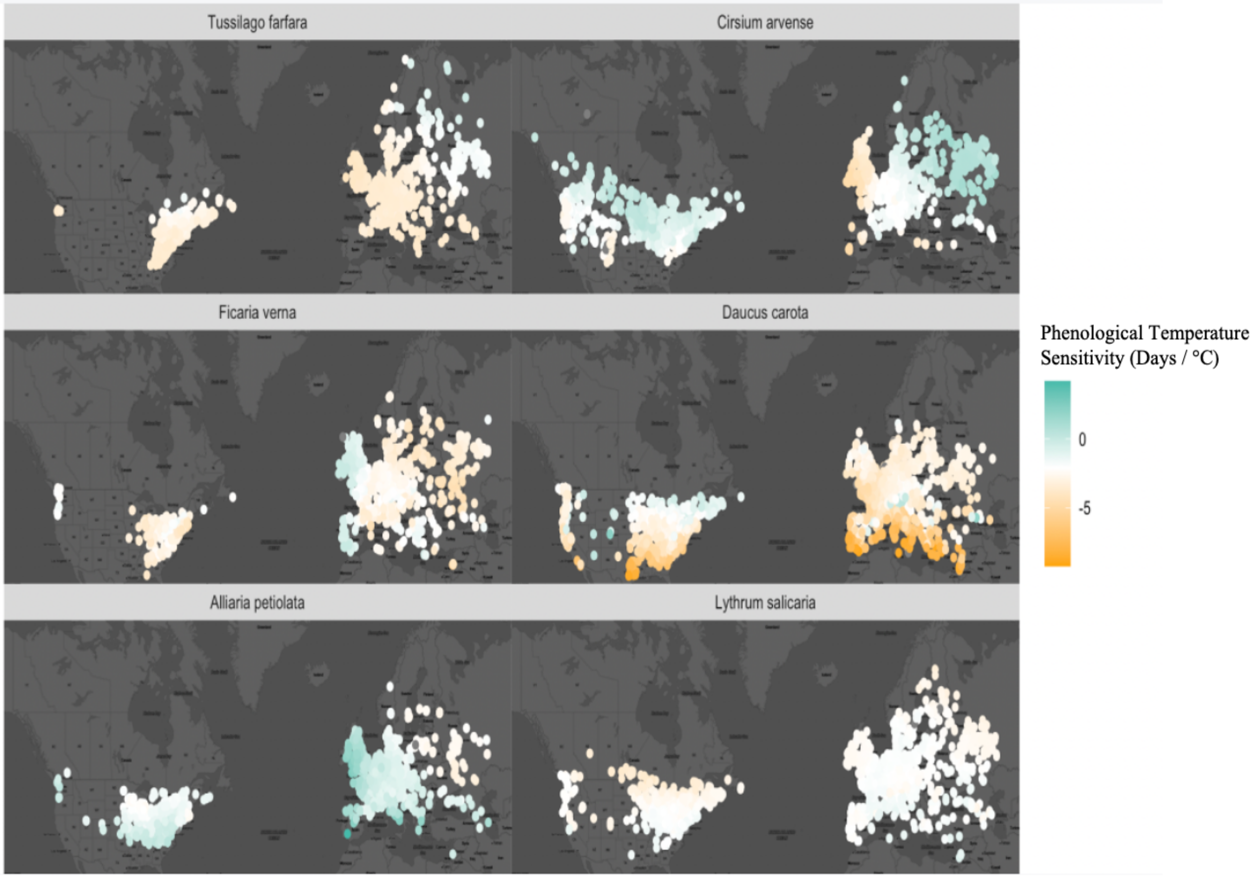

Figure S3 Geographic map of species-specific intraspecific variation in phenological temperature sensitivity. Points represent geolocated species observations from iNaturalist and color value represents the estimated temperature sensitivity of plants in that location, based on the local value of MAT and IVT. Color scale is centered on overall mean temperature sensitivity (-2.28 days / °C), with teal indicating a weaker-than-average level of temperature sensitivity and orange indicating a stronger-than-average level of temperature sensitivity. Temperature sensitivity values are estimated marginal trends, extracted across all unique combinations of species identity, MAT, and IVT (n = 23,220, df = 23,100, adjusted R^2 = 0.817).

Supplementary Tables

Table S1. Description of life history species traits for six herbaceous plant species included in this study. [1, 2, 3, 4, 5, 6] Under "Primary Habitat", "Forest" refers to deciduous temperate forest habitat, "Old Field" refers to open disturbed habitat such as agricultural fields, roadsides, and meadows, "Mesic Forest" refers to forest habitat that is moderately saturated by moisture, "Mesic Old Field" describes open disturbed habitat that is moderately saturated by moisture, "Wetland" describes open habitat that is heavily saturated by moisture such as streambanks, marshes, and lakeshores. Under "Reason(s) of introduction in North America", "Horticulture" describes garden species which were cultivated for culinary purposes, "Medicinal" refers to species cultivated for medical / healing purposes, "Accidental" refers to unintentional introductions. "Ornamental" refers to species cultivated for decorative or landscaping use.

**Species**	**Family**	**Primary habitat**	**Life History**	**Time of introduction in North America (estimated)**	**Reason(s) of introduction in North America (estimated)**	**Seasonal timing of reproduction (approx.)**
Alliaria petiolata	Brassicaceae	Forest	Biennial	1800s	Horticulture and Medicinal	Mid-spring
Cirsium arvense	Asteraceae	Old Field	Perennial	1600s	Accidental	Summer
Daucus carota	Apiaceae	Old Field	Biennial	1600s	Medicinal and/or Accidental	Summer
Ficaria verna	Ranunculaceae	Mesic Forest	Perennial	1860s	Ornamental	Early-spring
Lythrum salicaria	Lythraceae	Wetland	Perennial	1800s	Ornamental and Medicinal	Summer
Tussilago farfara	Asteraceae	Mesic Old Field	Perennial	1840s	Medicinal	Early-spring

Table S2. Sample sizes of final dataset across species and continents.

Species name	(n) reproductive phenology observations in Eurasia	(n) reproductive phenology observations in North America
Alliaria petiolata	2617	2353
Cirsium arvense	1666	1589
Daucus carota	2030	2031
Ficaria verna	2160	1868
Lythrum salicaria	2071	2020
Tussilago farfara	1366	1418

Table S3: Model selection for the 'global model' to assess intraspecific variation in 'temperature sensitivity' (Aims 2 and 3). During preliminary model selection, we identified additional factors that were not directly relevant to the study questions but substantially improved the explanatory power of the Global model ('standardized precipitation', 'species identity', and 'phenophase'; A). Model fit was optimized by defining these factors as fixed interaction effects, rather than as random effects or as fixed effects without interactions (B-E). We also evaluated the possibility that multi-way interactions unnecessarily increased the complexity of the global model by conducting stepwise backwards selection. Using the 'stepAIC' function from the 'MASS' package in R[7], we systematically dropped model terms which did not improve AIC value (F). The backward-selected model retained the majority of terms, including the interaction terms reported in the main results: (standardized temperature * MAT * IVT), and (standardized temperature * species). A handful of higher-order interaction terms associated with 'standardized precipitation' and 'phenophase', which were not of primary interest to the analysis, were dropped in favor of lower-order interactions terms. Given the elevated simplicity of the original model structure (A vs. F), we chose retain the "Global model" (A) over the backward-selected model (F) for our study.

Model structure	AIC
**A. "Global model" (as seen in the main text):**<br><br>DOY ~<br>standardized temperature*IVT*MAT*species<br>+ standardized precipitation*IVT*MAT*species<br>+ phenophase*IVT*MAT*species	215229.3
**B. Model lacking 'species' as an interaction term**<br><br>DOY ~<br>standardized temperature*IVT*MAT<br>+ standardized precipitation*IVT*MAT<br>+ phenophase*IVT*MAT<br>+ species	216506.7
**C. Model lacking 'phenophase' as an interaction term**<br><br>DOY ~<br>standardized temperature*IVT*MAT*species<br>+ standardized precipitation*IVT*MAT*species<br>+ phenophase	215543.8
**D. Model lacking 'standardized precipitation' as an interaction term**<br><br>DOY ~<br>standardized temperature*IVT*MAT*species<br>+ phenophase*IVT*MAT*species<br>+ standardized precipitation	215349.4

**E. Model including 'species' and 'phenophase' as random effects**<br><br>DOY ~<br>standardized temperature*IVT*MAT<br>+ standardized precipitation*IVT*MAT<br>+ (1|species)<br>+ (1|phenophase)	216698.4
**F. Backwards selection on the "Global model":**<br><br>DOY ~<br>standardized temperature<br>+ IVT<br>+ MAT<br>+ species<br>+ standardized precipitation<br>+ phenophase<br>+ standardized temperature* IVT<br>+ standardized temperature* MAT<br>+ IVT*MAT<br>+ standardized temperature*species<br>+ IVT*species<br>+ MAT*species<br>+ IVT*standardized precipitation<br>+ MAT*standardized precipitation<br>+ species*standardized precipitation<br>+ IVT*phenophase<br>+ MAT*phenophase<br>+ species*phenophase<br>+ standardized temperature*IVT*MAT<br>+ standardized temperature*IVT*species<br>+ standardized temperature*MAT*species<br>+ IVT*MAT*species<br>+ IVT*MAT*standardized precipitation<br>+ IVT*species*standardized precipitation<br>+ MAT*species*standardized precipitation<br>+ IVT*MAT*phenophase<br>+ IVT*species*phenophase<br>+ standardized temperature*IVT*MAT*species	215208.3

Table S4. Species-specific estimates of in temperature sensitivity (measured as the marginal effect of standardized temperature on phenology DOY) and intraspecific variation in temperature sensitivity (measured as the difference between the maximum and minimum marginal estimates of temperature sensitivity across the study region, with MAT ranging -5 to +15 °C and IVT ranging 0.4 to 1.2 s.d.) (n = 23,220, df = 23,100, adjusted $R^2 = 0.817$).

Species Identity	Temperature Sensitivity (days/°C)	Standard Error	p value	Intraspecific Range in Temperature Sensitivity (min – max, days/°C)
All species	-2.28	0.13	< 0.001 **	3.12
T. farfara	-3.37	0.32	< 0.001 **	5.25
F. verna	-2.78	0.28	< 0.001 **	11.74
A. petiolata	-1.07	0.24	< 0.001 **	13.98
C. arvense	-1.52	0.37	< 0.001 **	9.11
D. carota	-2.87	0.31	<0.001 **	16.4
L. salicaria	-2.05	0.34	<0.001 **	4.89

Table S5. Species-specific independent effects of mean annual minimum temperature (MAT) and interannual variability in temperature (IVT) on temperature sensitivity (measured as the marginal effect of standardized temperature on phenology DOY). Model estimates control for variation in phenophase and standardized precipitation (n = 23,220, df = 23,100, adjusted $R^2 = 0.817$).

Species identity	Effect of Mean Annual Temperature (Δ temperature sensitivity / °MAT)	Standard Error	p value	Effect of Interannual Temperature Variability (Δ temperature sensitivity / s.d. IVT)	Standard Error	p value
All Species	-0.10	0.02	<0.001**	-0.9	0.85	0.529
T. farfara	-0.05	0.07	0.723	2.23	2.13	0.547
F. verna	-0.26	0.07	<0.001**	-7.28	1.58	<0.001**
A. petiolata	0.06	0.06	0.590	-7.15	1.50	<0.001**
C. arvense	0.01	0.04	0.941	8.17	2.25	<0.001**
D. carota	-0.50	0.04	<0.001**	-2.75	2.42	0.495
L. salicaria	0.14	0.05	0.014	1.38	2.23	0.814

Table S6. Species-specific marginal estimates and contrasts in temperature sensitivity (measured as the effect of standardized temperature on phenology DOY) across interacting levels of mean annual temperature (MAT) and interannual variability in temperature (IVT). The marginal estimates displayed here are subsampled at (-5 and +15 °C MAT) and (0.4 and 1.2 s.d. IVT). Marginal estimates of temperature sensitivity control for variation in species identity, phenophase, and standardized precipitation (n = 23,220, df = 23,100, adjusted $R^2 = 0.817$).

IVT (s.d.)	Species Identity	MAT (C)	Contrast	Temp. Sensitivity	SE	lower.CL	upper.CL
0.4	Tussilago farfara	-5	.	-5.65989834	1.8402982	-11.5439546	0.2241579
0.4	Tussilago farfara	15	.	-2.80479182	2.1983210	-9.8335681	4.2239844
1.2	Tussilago farfara	-5	.	-0.40536174	1.1859805	-4.1973428	3.3866193
1.2	Tussilago farfara	15	.	-4.90168658	1.5673256	-9.9129570	0.1095838
0.4	Ficaria verna	-5	.	-0.01218701	1.3110587	-4.2040854	4.1797114
0.4	Ficaria verna	15	.	0.62025688	1.4210342	-3.9232705	5.1637842
1.2	Ficaria verna	-5	.	-0.53641426	1.3068718	-4.7149257	3.6420972
1.2	Ficaria verna	15	.	-11.12368344	1.4873892	-15.8793701	-6.3679968
0.4	Alliaria petiolata	-5	.	-2.54262259	1.8334504	-8.4047843	3.3195391
0.4	Alliaria petiolata	15	.	6.94601358	2.0156938	0.5011583	13.3908688
1.2	Alliaria petiolata	-5	.	-0.85872707	1.3087261	-5.0431673	3.3257132
1.2	Alliaria petiolata	15	.	-7.03826062	1.7038503	-12.4860466	-1.5904746
0.4	Cirsium arvense	-5	.	-3.87820393	1.0944701	-7.3775955	-0.3788124
0.4	Cirsium arvense	15	.	-6.17605245	1.5786501	-11.2235310	-1.1285739
1.2	Cirsium arvense	-5	.	0.34620634	0.8268454	-2.2974983	2.9899110
1.2	Cirsium arvense	15	.	2.94101166	1.7959348	-2.8011997	8.6832231
0.4	Daucus carota	-5	.	6.03674906	1.6206197	0.8550793	11.2184189
0.4	Daucus carota	15	.	-10.36623292	1.0599180	-13.7551495	-6.9773163
1.2	Daucus carota	-5	.	-1.55669628	1.4052516	-6.0497612	2.9363687
1.2	Daucus carota	15	.	-6.52817249	1.4039739	-11.0171523	-2.0391926
0.4	Lythrum salicaria	-5	.	-2.84984309	1.5299644	-7.7416572	2.0419711
0.4	Lythrum salicaria	15	.	-2.36217265	1.3124935	-6.5586585	1.8343132
1.2	Lythrum salicaria	-5	.	-3.83809537	0.9461462	-6.8632450	-0.8129457
1.2	Lythrum salicaria	15	.	1.05098382	1.4582970	-3.6116854	5.7136531
0.4	Tussilago farfara	.	(-5) - 15	-2.85510651	3.4578114	-13.9108999	8.2006868

**1.2**	**Tussilago farfara**	**.**	**(-5) - 15**	**4.49632484**	**2.2961485**	**-2.8452390**	**11.8378887**
**0.4**	**Ficaria verna**	**.**	**(-5) - 15**	**-0.63244389**	**2.3108280**	**-8.0209432**	**6.7560554**
**1.2**	**Ficaria verna**	**.**	**(-5) - 15**	**10.58726918**	**2.4638457**	**2.7095205**	**18.4650178**
**0.4**	**Alliaria petiolata**	**.**	**(-5) - 15**	**-9.48863617**	**3.5899020**	**-20.9667681**	**1.9894958**
**1.2**	**Alliaria petiolata**	**.**	**(-5) - 15**	**6.17953356**	**2.7748670**	**-2.6926556**	**15.0517227**
**0.4**	**Cirsium arvense**	**.**	**(-5) - 15**	**2.29784852**	**1.7229300**	**-3.2109419**	**7.8066389**
**1.2**	**Cirsium arvense**	**.**	**(-5) - 15**	**-2.59480532**	**2.0431312**	**-9.1273874**	**3.9377767**
**0.4**	**Daucus carota**	**.**	**(-5) - 15**	**16.40298198**	**1.8992623**	**10.3303976**	**22.4755664**
**1.2**	**Daucus carota**	**.**	**(-5) - 15**	**4.97147621**	**1.9295743**	**-1.1980259**	**11.1409784**
**0.4**	**Lythrum salicaria**	**.**	**(-5) - 15**	**-0.48767044**	**1.9681120**	**-6.7803908**	**5.8050500**
**1.2**	**Lythrum salicaria**	**.**	**(-5) - 15**	**-4.88907919**	**1.7127645**	**-10.3653670**	**0.5872086**

Table S7. Estimated marginal trends in precipitation sensitivity (measured as the effect of standardized precipitation on phenology DOY) across interacting levels of mean annual temperature and interannual variability in temperature (IVT). Estimates of precipitation sensitivity are subsampled at -5, 5, and 15 °C MAT and at 0.4, 0.8, and 1.2 s.d. IVT. Marginal estimates of precipitation sensitivity control for variation in species identity, phenophase, and standardized temperature (n = 23,220, df = 23,100, adjusted R^2 = 0.817).

**Mean Annual Temperature (°C)**	**Interannual Variability in temperature (s.d.)**	**Phenological Precipitation Sensitivity (days/mm)**	**Standard Error**	**p value**
Overall	Overall	0.04	0.01	<0.001**
-5	0.4	-0.09	0.09	0.311
	0.8	0.08	0.04	0.034**
	1.2	0.26	0.09	0.001**
5	0.4	-0.02	0.03	0.450
	0.8	0.03	0.01	0.004**
	1.2	0.08	0.03	0.014**
15	0.4	0.05	0.10	0.636
	0.8	-0.02	0.04	0.646
	1.2	-0.09	0.12	0.477

Table S8. Estimated marginal trends in precipitation sensitivity (measured as the effect of standardized precipitation on phenology DOY) across a gradient of mean annual temperature (MAT) and at different levels of interannual variability in temperature (IVT). The marginal effect of MAT on precipitation sensitivity (**Δ** temperature sensitivity / °MAT) is estimated across a range of -5 to 15 °C MAT, and subsampled at 0.4, 0.8, and 1.2 s.d. IVT. Model estimates control for variation in species identity, phenophase, and standardized temperature (n = 23,220, df = 23,100, adjusted R^2 = 0.817).

**Interannual Temperature Variability (s.d.)**	**Effect of Mean Annual Temperature (Δ temperature sensitivity / °MAT)**	**Standard Error**	**p value**
Overall	-0.005	0.004	0.410
0.4	0.007	0.009	0.720
0.8	-0.005	0.004	0.410
1.2	-0.017	0.010	0.222

Table S9. Species-specific estimated marginal trends in precipitation sensitivity (measured as the mean effect of standardized precipitation on phenology DOY). Marginal estimates control for variation in phenophase, standardized temperature, MAT, and IVT (n = 23,220, df = 23,100, adjusted R^2 = 0.817).

**Species Identity**	**Phenological Precipitation Sensitivity (days/mm)**	**Standard Error**	**p value**
All species	0.03	0.01	<0.001**
T. farfara	-0.02	0.04	0.689
F. verna	0.09	0.02	<0.001**
A. petiolata	0.10	0.02	<0.001**
C. arvense	-0.13	0.02	<0.001**
D. carota	0.09	0.02	<0.001**
L. salicaria	0.05	0.02	<0.001**

References

1. Invasive.org. https://www.invasive.org/browse/subinfo.cfm?sub=3005. Accessed 11/4/2022.
2. Invasive.org. https://www.invasive.org/alien/pubs/midatlantic/ciar.htm#:~:text=Twenty%20large%20national%20parks%20across,not%20very%20tolerant%20of%20shade. Accessed 11/4/2022.
3. CABI. https://www.cabi.org/isc/datasheet/18018. Accessed 11/4/2022.
4. Brandywine Conservancy. https://www.brandywine.org/conservancy/blog/invasive-species-spotlight-lesser-celandine-ficaria-verna. Accessed 11/4/2022.
5. Invasive.org. https://www.invasive.org/alien/pubs/midatlantic/lysa.htm. Accessed 11/4/2022.
6. USDA FEIS. https://www.fs.usda.gov/database/feis/plants/forb/tusfar/all.html. Accessed 11/4/2022.
7. Venebles WN, Ripley BD (2002). *Modern Applied Statistics with S*, Fourth edition. Springer, New York. ISBN 0-387-95457-0, https://www.stats.ox.ac.uk/pub/MASS4/.
